# Identification of Molecular Pathologies Sufficient to Cause Neuropathic Excitability in Primary Somatosensory Afferents Using Dynamical Systems Theory

**DOI:** 10.1371/journal.pcbi.1002524

**Published:** 2012-05-24

**Authors:** Young-Ah Rho, Steven A. Prescott

**Affiliations:** Department of Neurobiology and the Pittsburgh Center for Pain Research, University of Pittsburgh, Pittsburgh, Pennsylvania, United States of America; École Normale Supérieure, College de France, CNRS, France

## Abstract

Pain caused by nerve injury (*i.e.* neuropathic pain) is associated with development of neuronal hyperexcitability at several points along the pain pathway. Within primary afferents, numerous injury-induced changes have been identified but it remains unclear which molecular changes are necessary and sufficient to explain cellular hyperexcitability. To investigate this, we built computational models that reproduce the switch from a normal spiking pattern characterized by a single spike at the onset of depolarization to a neuropathic one characterized by repetitive spiking throughout depolarization. Parameter changes that were sufficient to switch the spiking pattern also enabled membrane potential oscillations and bursting, suggesting that all three pathological changes are mechanistically linked. Dynamical analysis confirmed this prediction by showing that excitability changes co-develop when the nonlinear mechanism responsible for spike initiation switches from a quasi-separatrix-crossing to a subcritical Hopf bifurcation. This switch stems from biophysical changes that bias competition between oppositely directed fast- and slow-activating conductances operating at subthreshold potentials. Competition between activation and inactivation of a single conductance can be similarly biased with equivalent consequences for excitability. “Bias” can arise from a multitude of molecular changes occurring alone or in combination; in the latter case, changes can add or offset one another. Thus, our results identify pathological change in the nonlinear interaction between processes affecting spike initiation as the critical determinant of how simple injury-induced changes at the molecular level manifest complex excitability changes at the cellular level. We demonstrate that multiple distinct molecular changes are sufficient to produce neuropathic changes in excitability; however, given that nerve injury elicits numerous molecular changes that may be individually sufficient to alter spike initiation, our results argue that no single molecular change is necessary to produce neuropathic excitability. This deeper understanding of degenerate causal relationships has important implications for how we understand and treat neuropathic pain.

## Author Summary

Neuropathic pain results from damage to the nervous system. Much is known about the multitude of molecular and cellular changes that are triggered by nerve injury (and which *correlate* with development of neuropathic pain), but little is understood about how those changes *cause* neuropathic pain. Rather than identifying what changes occur after nerve injury (which has already been the focus of countless studies), our study focuses on identifying which changes are functionally important. Specifically, we explain how certain molecular changes, acting alone or in combination, cause a triad of neuropathic changes in primary afferent excitability. Through computational modeling and nonlinear dynamical analysis, we demonstrate that the entire triad of excitability changes arises from a single switch in the nonlinear mechanism responsible for spike initiation. Going further, we demonstrate that *many distinct molecular changes are sufficient* to produce that switch but that *no single molecular change is necessary* if more than one sufficient change co-occurs after nerve injury, which appears to be the case. The issue becomes whether molecular changes combine to reach some tipping point whereupon cellular excitability is qualitatively altered. This highlights the importance of nonlinearities for neuropathic pain and the need for more computational pain research.

## Introduction

**Figure 1:**
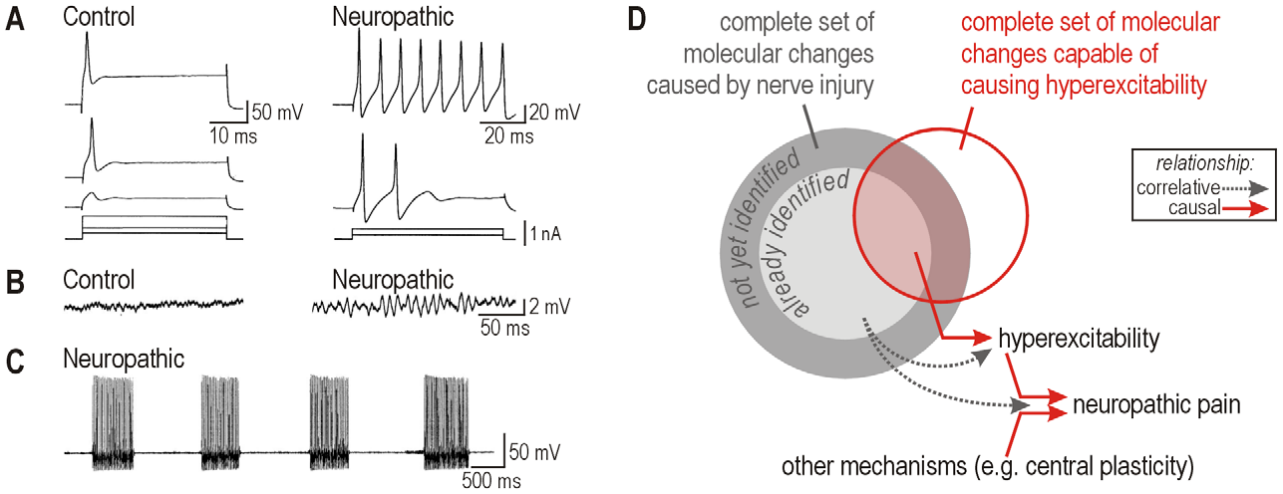
Neuropathic changes in primary afferent excitability. (**A**) Sample responses from large diameter acutely isolated dorsal root ganglion (DRG) neurons under control conditions (*normal*) and two days after L5 spinal nerve transection (*neuropathic*). Spiking pattern switches from onset-only to repetitive. (**B**) Sample responses, with the same average membrane potential of −36 mV, showing development of membrane potential oscillations (MPOs) after nerve injury. (**C**) Sample response showing bursting after nerve injury. (**D**) Venn diagram distinguishing classes of molecular changes and their relationship to primary afferent hyperexcitability and neuropathic pain. In this study, we sought to define the red circle. Parts A–C were modified from reference 4.

Many primary afferents become hyperexcitable after nerve injury. The resulting spontaneous and evoked hyperactivity contributes to neuropathic pain directly and by driving central sensitization [1], [2], [3]. Beyond simply becoming *more* excitable (*i.e.* having a lower activation threshold), three qualitative changes in excitability stand out: a change in spiking pattern (**Fig. 1A**), membrane potential oscillations (**Fig. 1B**) and bursting (**Fig. 1C**) [4], [5], [6], [7], [8], [9], [10], [11], [12]. These changes co-occur and have been documented in dorsal root ganglion (DRG) neurons of various sizes, including putative high- and low-threshold afferents. Hyperexcitability in low-threshold afferents is thought to underlie allodynia [5], [13], [14], [15], which implicates central plasticity (*e.g.* unmasking of polysynaptic spinal circuits through disinhibition [16], [17]) such that normally innocuous stimulation (causing exaggerated responses among hyperexcitable low-threshold afferents) can lead to activation of ascending pain pathways, thus enabling innocuous stimulation to elicit pain. The central nervous system is obviously required for pain perception, and central plasticity certainly contributes to the development of neuropathic pain, but it is generally agreed that reversing peripheral hyperexcitability could relieve or markedly attenuate many varieties of neuropathic pain [3]. Doing so has proven easier said than done.

Countless molecular changes have been documented to occur after nerve injury and are correlated with cellular hyperexcitability and pain [[for reviews], [ see 18], [19,20]. Still more injury-induced changes are likely to occur but have yet to be described (**Fig. 1D**). Moreover, causal relationships are harder to ascertain than correlations. Knockout studies [e.g. 21], [22] can demonstrate the necessity of certain molecules for mediating changes in cellular excitability, but those studies do not address sufficiency. If control of primary afferent excitability is degenerate, meaning distinct molecular changes yield equivalent cellular outcomes [23], then a certain molecular change may be sufficient but unnecessary to produce hyperexcitability. Specifically, if more than one molecular change is sufficient to cause cellular hyperexcitability, then blocking any one of those molecular changes will not prevent hyperexcitability if another molecular change can fill in. This possibility should be cause for alarm given that multiple changes co-occur following nerve injury (see above), yet this has not been considered in recent discussions on the lack of progress in translational pain research [24], [25].

Thus, beyond considering which molecular changes are necessary, it is also important (1) to identify which molecular changes are sufficient to produce cellular hyperexcitability and why, and (2) to explain how co-occurring molecular changes interact. Addressing these issues is complicated by the complex nature of excitability. Complexity stems from nonlinearities, which is to say that components of a system (*e.g.* ion channels within a neuron) compete, cooperate, or interfere with one another [26]. A nonlinear system is not the sum of its parts, which means reductionist approaches provide incomplete, if not incorrect, explanations of complex phenomena. Dynamical systems theory provides a more integrative approach but has not been used hitherto to help explain primary afferent hyperexcitability.

In this study, we sought to identify what sorts of molecular changes could, in theory, cause primary afferent hyperexcitability. This is fundamentally different from identifying what changes occur after nerve injury. The two approaches are complementary (**Fig. 1D**), but whereas the latter has seen widespread use, the former has not. Hence, the current study provides a novel perspective that should facilitate the interpretation of past results. Rather than simulating known molecular changes, we worked in the reverse direction by first reproducing known changes in cellular excitability and then using theory to help identify their potential molecular bases. Specifically, we reproduced previously reported [4] injury-induced changes in cellular excitability (see **Fig. 1A–C**) in the simplest possible conductance-based model. Then, through dynamical analysis of that model, we pinpointed a switch in spiking mechanism as the common dynamical basis for all three excitability changes. In turn, we investigated the potential molecular bases for the switch in spiking mechanism. As anticipated, several distinct molecular changes were found to produce qualitatively identical changes in cellular excitability (by switching the spike initiation mechanism), thus highlighting degeneracy in the molecular basis for cellular excitability. We also found that molecular changes can add together and offset one another in terms of their influence on excitability. The implications of these insights for understanding and treating neuropathic pain are discussed.

## Results

### Injury-induced change in spiking pattern

**Figure 2:**
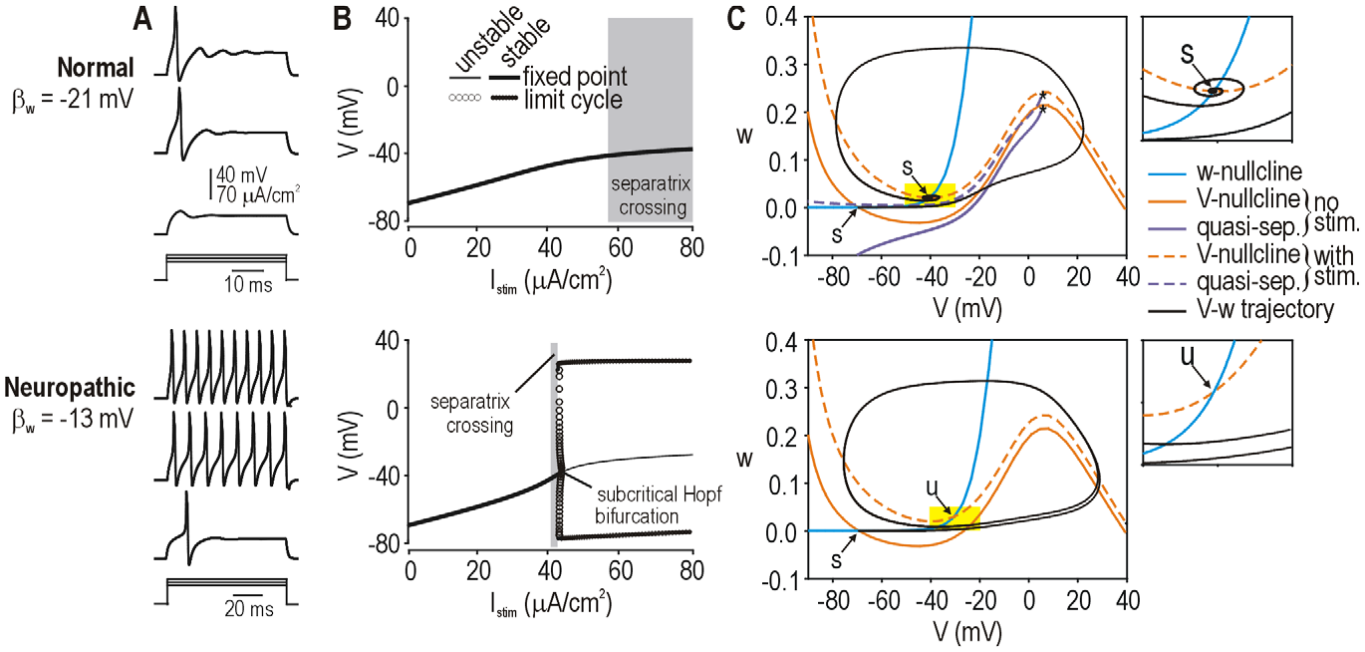
Simulation and dynamical explanation of change in spiking pattern. (**A**) Spiking pattern during sustained depolarization was converted from onset-only (normal, β_w_ = −21 mV) to repetitive (neuropathic, β_w_ = −13 mV) by varying a single parameter. Onset-only spiking was observed in the neuropathic model but for only a narrow stimulus range. (**B**) According to bifurcation analysis in which stimulation (*I*_stim_) was systematically varied, repetitive spiking was produced by the neuropathic model when *I*_stim_ exceeded a critical value required for a subcritical Hopf bifurcation. In contrast, the normal model did not undergo a bifurcation, which means spiking was limited to single spikes generated through a QS-crossing (see below). Generation of a single spike does not constitute a change in steady-state behavior, consistent with the absence of a bifurcation. (**C**) Phase planes show the fast activation variable *V* plotted against the slower recovery variable *w*. Nullclines (color) indicate where *V* or *w* do not change. Excitatory stimulation shifts the *V*-nullcline upward without affecting the *w*-nullcline. In the neuropathic model, *V*- and *w*-nullclines intersect at a stable (*s*) fixed point prior to stimulation, but that point becomes unstable (*u*) during stimulation – this corresponds to a Hopf bifurcation and is responsible for repetitive spiking. In the normal model, the fixed point remains stable during stimulation despite the *V*-nullcline shifting upward, but a single spike can nonetheless be generated depending on how the system moves to the newly positioned fixed point. The trajectory can be predicted by reference to a quasi-separatrix (QS), which corresponds to a manifold in phase space from which trajectories diverge. Quasi-separatrices were plotted here by integrating with a negative time step with initial values indicated by * on the phase planes (see Methods). Like the *V*-nullcline, the QS shifts instantaneously with stimulation. If, as shown, the original fixed point ends up below the shifted QS, the trajectory to the newly positioned fixed point must follow an indirect route around the end of the QS (*), thus producing a spike; a more direct, subthreshold route would require the trajectory to cross back over the QS, which is not possible. If the original fixed point remained above the shifted QS, the trajectory would follow a direct route and no spike would be produced (not illustrated).

The first of three pathological changes that we sought to reproduce in our computer model was the switch from onset-only spiking to repetitive spiking during sustained depolarization (see **Fig. 1A**). Starting with a minimal conductance-based model (see Eqn. 1–5 in Methods), we set parameters in order for the model to produce onset-only spiking (**Fig. 2A** **top**) and then we varied parameters one at a time in order to switch the model to repetitive spiking (**Fig. 2A** **bottom**). We initially focused on the effects of varying parameter β_w_, and we henceforth refer to the model with β_w_ = −21 mV as “normal” and to the model with β_w_ = −13 mV as “neuropathic”. The biophysical meaning of a change in β_w_ and the effects of varying other parameters (in the same model or in different models) are considered later in the Results.

Based on our past work [27], we hypothesized that each spiking pattern was associated with a dynamically distinct mechanism of spike initiation. To test this, we conducted bifurcation analysis in the normal and neuropathic models. A bifurcation refers to a qualitative change in steady-state behavior, *e.g.* transition from quiescence to repetitive spiking. Bifurcation analysis involves systematically varying a parameter of interest to determine at what value(s) of that parameter the system switches steady states, and how that switch occurs. When we varied the stimulus current *I*_stim_, the neuropathic model transitioned from quiescence to repetitive spiking through a subcritical Hopf bifurcation (**Fig. 2B** **bottom**) whereas the normal model exhibited no bifurcation (**Fig. 2B** **top**). Both models exhibited a range of *I*_stim_ within which a single, onset-only spike was generated independently of a bifurcation (see below).

We further characterized spike initiation using phase plane analysis. Our starting model comprised just two variables, *V* and *w*, whose interaction is entirely responsible for how the model behaves. Those interactions can be visualized by plotting *V* against *w* to create a phase plane, and can be analyzed by considering how the nullclines intersect, where *V*- and *w*-nullclines represent locations in phase space where *V* or *w* do not change. Points where the two nullclines intersect, referred to as fixed points, are especially important for explaining spike initiation. Stimulation caused a vertical shift in the *V*-nullcline, which, in the neuropathic model, caused the stable fixed point to become unstable (**Fig. 2C** **bottom**) – this is the geometric explanation for the Hopf bifurcation responsible for repetitive spiking. In the normal model, stimulation caused a similar shift in the *V*-nullcline, which allowed one spike to be generated despite the fixed point remaining stable (**Fig. 2C** **top**). Rather than occurring through a bifurcation, spike generation in the latter case depended on the trajectory to the newly positioned (but still stable) fixed point, as explained here. A quasi-separatrix (QS) corresponds a manifold in phase space from which trajectories diverge. The *V*-nullcline and QS shift instantaneously upon the onset of stimulation. Although the QS can move, trajectories cannot cross a stationary QS and, instead, tend to diverge from it; therefore, if the QS shifts far enough during stimulation that the starting point of the trajectory (which corresponds to the original fixed point) ends up below the shifted QS, the system will move to its new fixed point via an indirect route around the end of the QS. See Prescott et al. [27] for more detailed explanations of these dynamical mechanisms.

The biophysical meaning of these dynamical mechanisms in terms of competition between fast-activating inward current (a positive feedback process) and slower-activating outward current (a negative feedback process) are explained in **Fig. S1**. Equivalent changes can be observed in other minimal 2-D models, for instance, if delayed rectifier potassium channel activation is replaced with fast sodium channel inactivation (**Fig. S2**).

### Injury-induced development of membrane potential oscillations (MPOs)

The second of three pathological changes that we sought to reproduce in our computer model was the development of MPOs (see **Fig. 1B**). We hypothesized that the same dynamical mechanism responsible for repetitive spiking also explains MPOs because the fixed point identified in **Fig. 2C** is a focus (*i.e.* it has complex eigenvalues), which means trajectories spiral into or away from that point depending on whether the point is stable or unstable, respectively (see inset of **Fig. 2C** **top** for sample trajectory). Any noise within the system (e.g. channel noise [28]; primary afferent somata bear no synapses, thus excluding synaptic noise) will continuously perturb the system away from its stable fixed point; as the system relaxes, it will return to the fixed point via a spiral trajectory, thereby producing oscillations on the time series [29]. Thus, one would expect noise-dependent MPOs whose amplitude is inversely proportional to the stability of the fixed point. Since a Hopf bifurcation represents destabilization of the fixed point (see above), noise-dependent MPOs should become prominent near the bifurcation. In biophysical terms, this corresponds to inward current starting to activate but activation of outward current (or inactivation of inward current) catching up, thus preventing spike initiation but producing an MPO in the process.

**Figure 3:**
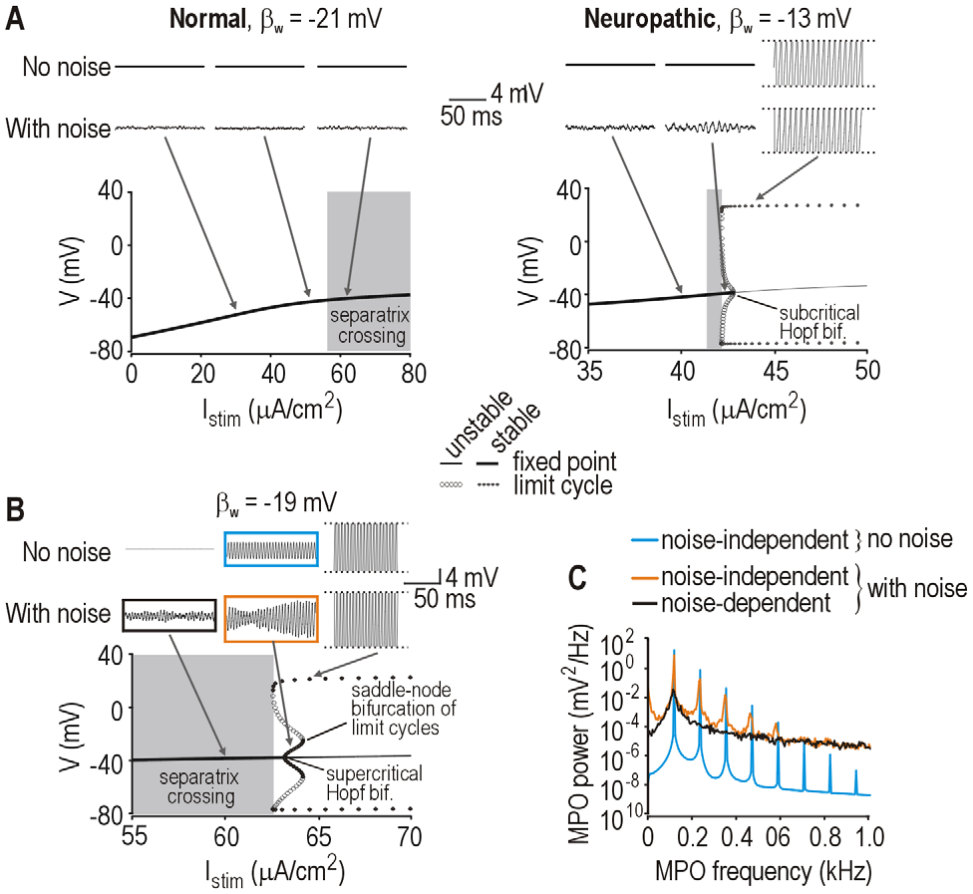
Simulation and dynamical explanation of membrane potential oscillations. (**A**) Traces (*top*) show sample responses for different *I*_stim_ indicated along the bifurcation diagrams (*bottom*). One set of simulations included noise (see Methods). In the normal model, MPOs were negligible regardless of noise conditions. In the neuropathic model, noise-dependent MPOs were present and were most prominent for *I*_stim_ near the bifurcation, when the fixed point is nearly unstable. Noise-dependent MPOs are not associated with a stable limit cycle. (**B**) In model with β_w_ = −19 mV, noise-independent MPOs occurred because a supercritical Hopf bifurcation produced a stable, subthreshold limit cycle. The stable limit cycle existed for only a narrow range of *I*_stim_ before being destroyed through a saddle-node bifurcation of limit cycles. Noise-dependent MPOs still occurred for *I*_stim_ just below the supercritical Hopf bifurcation. Boxed traces show conditions analyzed in C. (**C**) Power spectra comparing noise-dependent and -independent MPOs with and without noise. Colors correspond to boxes in B. Broadly peaked power spectra (like those associated with MPOs under noisy conditions) more closely resemble experimental data (see Results).

Based on these theoretical insights, we made three predictions: (1) noise-dependent MPOs should occur in the neuropathic model when it operates near a subcritical Hopf bifurcation, (2) noise-dependent MPOs should not occur in the normal model because there is no Hopf bifurcation, (3) noise-*in*dependent MPOs should not occur in either model because there is no stable, subthreshold limit cycle on the bifurcation diagrams in **Fig. 2B**. To test these predictions, we ran simulations with and without noise in the normal and neuropathic 2-D models (without having adjusted any model parameters). All three predictions were confirmed (**Fig. 3A**). These results argue that repetitive spiking and MPOs are two manifestations of the same dynamical mechanism, namely a subcritical Hopf bifurcation, and become manifest when *I*_stim_ is above or just below the bifurcation point, respectively. In the absence of a Hopf bifurcation, the system can neither spike repetitively nor exhibit MPOs.

Previous efforts to model injury-induced MPOs in DRG neurons have not considered noise, and have thus necessarily focused on solutions involving stable subthreshold limit cycles [8], [30], [31]. To contrast noise-independent and -dependent MPOs, we adjusted our 2-D model to make it produce MPOs in the absence of any noise. In this model, MPOs arose from a stable limit cycle produced through a *super*critical Hopf bifurcation (**Fig. 3B**). Compared with noise-dependent MPOs, noise-independent MPOs were much larger and more regular as evidenced by large, narrow peaks on the power spectra (colored curves on **Fig. 3C**). Published power spectra of experimental MPOs are broad [5], [10], [30], like the black power spectrum in **Fig. 3C**, and are thus consistent with noise-dependent MPOs. Most published studies (see Introduction) have not included power spectral analysis, but the irregularity of MPOs in experimental time series points to a noise-dependent oscillatory mechanism.

**Figure 4:**
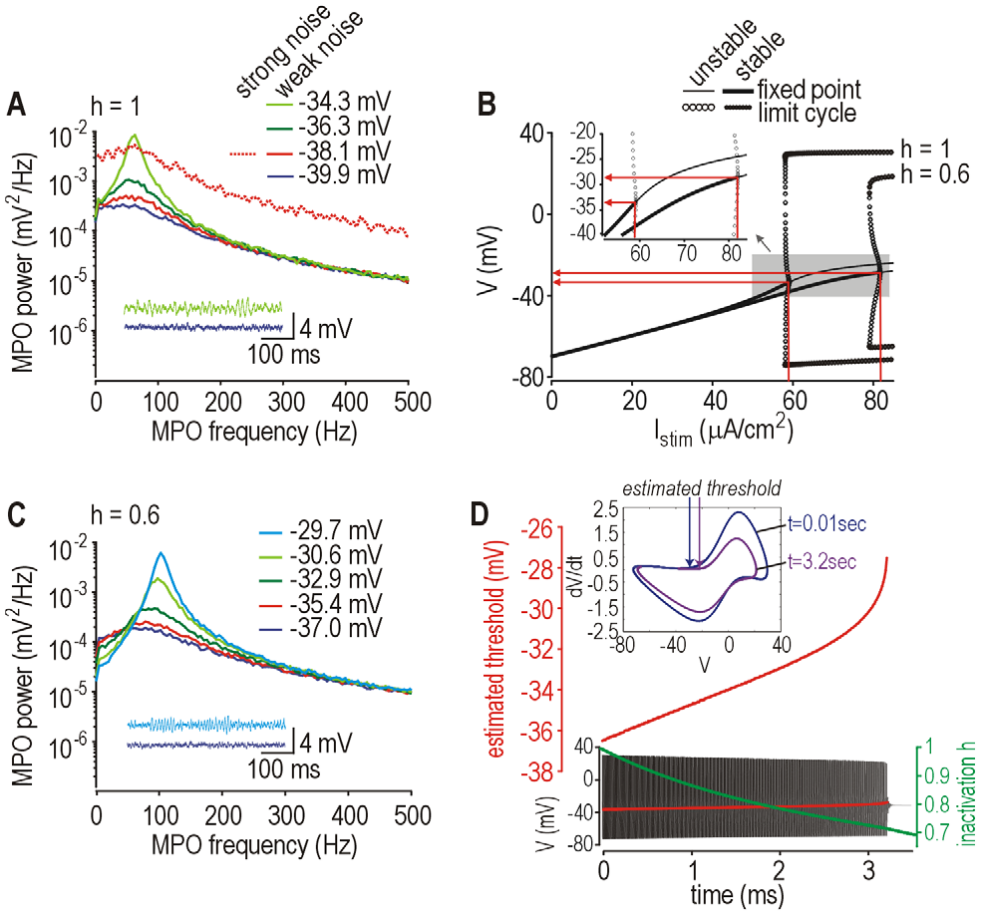
Frequency and voltage range of noise-dependent MPOs in neuropathic model. (**A**) Power spectra for MPOs at different average membrane potentials. Spike threshold was −33.4 mV according to bifurcation analysis. Noise-dependent MPOs occurred only within ∼4 mV of spike threshold. With 10× stronger noise (dotted line), the power spectrum was shifted upwards but was otherwise unaffected when compared against the weak noise condition (solid line) with equivalent mean depolarization. Peak frequency shifted slightly with mean depolarization. (**B**) Bifurcation diagrams show change in spike threshold caused by implementing cumulative Na^+^ inactivation, which is controlled by *h*. Diagrams are plotted for no inactivation (*h* = 1) and with 40% inactivation (*h* = 0.6). (**C**) Power spectra like in A but in a model with 40% Na^+^ channel inactivation. The shift in spike threshold (see B) allowed MPOs to occur at more depolarized potentials. (**D**) Change in spike threshold in a model that includes cumulative Na^+^ channel inactivation, controlled by *h*, as a third variable. Spike threshold was estimated as the voltage at the inflection point for *dV*/*dt*. Sample response shows progressive inactivation (decrease in *h*; *green*) and the corresponding rise in spike threshold (*red*) which eventually leads to the termination of repetitive spiking. Inset shows *dV*/*dt* vs. *V* for spike near the beginning and end of the spike train to highlight the change in spike shape and the shift in threshold.

Past experiments have shown that MPOs can occur over a broad range of membrane potentials, and that MPO amplitude and peak frequency tend to increase with mean depolarization [4], [10]. **Figure 4** demonstrates that our model can reproduce these MPO properties on the basis of a noise-dependent oscillatory mechanism. MPO amplitude grows as the average membrane potential approaches spike threshold (**Fig. 4A**). By comparison, for equivalent mean input, increasing noise amplitude shifts the power spectrum upward but does not change its shape (compare solid and dotted red curves), consistent with larger noisy perturbations but equivalent relaxation toward the fixed point; indeed, the imaginary part of the fixed point's complex eigenvalue, which reflects the rate of winding around the fixed point, is unchanged by noise. That said, stronger noise (which is arguably unphysiological in the case of DRG neurons and would overwhelm the intrinsic oscillatory process) increases the likelihood of the voltage trajectory crossing spike threshold; thus, probability of spike initiation depends jointly on noise amplitude and the difference between mean depolarization and voltage threshold [see 32]. If threshold is shifted (*e.g.* by slow cumulative sodium channel inactivation, which is represented here by *h*; **Fig. 4B**), the voltage range across which MPOs occur becomes quite broad and is associated with a shift in peak MPO frequency (**Fig. 4C**). Implementation of cumulative sodium channel inactivation as a dynamical process in our model (see Eqns. 6–8) demonstrates the feasibility of a large shift in spike threshold (**Fig. 4D**). Given that noise is ubiquitous, noise-dependent oscillations near a subcritical Hopf bifurcation provide a robust dynamical explanation of MPOs without invoking any mechanism beyond that already required to explain repetitive spiking.

### Injury-induced development of bursting

**Figure 5:**
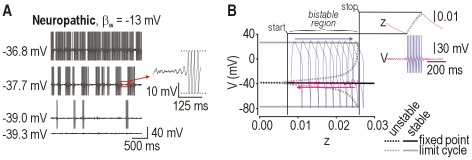
Simulation and dynamical explanation of bursting. (**A**) Sample responses at different average membrane potentials in the neuropathic model (β_w_ = −13 mV) with slow adaptation mediated by *I*_AHP_. Noise was included in all simulations and makes the bursting irregular (and thus more realistic) but noise is not necessary for bursting. Duration and frequency of bursts increased with average depolarization. (**B**) Bursting depends on hysteresis caused by bistability associated with the subcritical Hopf bifurcation. Inset shows *V* and *z* during sample burst, where *z* controls activation of *I*_AHP_. The same response, with its differently colored burst and interburst phases, was projected onto the bifurcation diagram created by treating *z* as a bifurcation parameter. The model tracks the stable limit cycle branch, spiking repetitively as *z* increases until the end of the branch is reached, at which point the burst stops. The model then tracks the stable fixed point as *z* decreases (during which noise-dependent MPOs wax and wane) until the fixed point becomes unstable, at which point another burst starts. Hysteresis is evident from the bursts starting and stopping at different values of *z*. This bifurcation diagram is flipped horizontally relative to those shown in other figures because the bifurcation parameter here controls *I*_AHP_, which is an inhibitory current, whereas *I*_stim_ (the bifurcation parameter used elsewhere) is excitatory.

The last of three pathological changes that we sought to reproduce in our model was the development of bursting (see **Fig. 1C**). Bursting is a slow process relative to the timescale of individual spikes. Because there is no variable with a sufficiently slow time constant in our 2-D model, we did not expect and nor did we observe bursting in that model. However, the subcritical Hopf bifurcation is known to allow elliptic bursting when a slow process, like spike frequency adaptation, causes the system to drift back and forth across the bifurcation [33], [34]. This occurs because a subcritical Hopf bifurcation has a region of bistability within which the neuron is quiescent or spiking depending on recent history (which is reflected in the adaptation) – this is an example of hysteresis. We reasoned, therefore, that adding adaptation to our neuropathic 2-D model (thus making it 3-D) should give rise to elliptic bursting, and that this might reproduce the bursting observed experimentally in nerve-injured DRG neurons. To test this, we added adaptation mediated through an AHP current *I*_AHP_ (see Eqns. 9 and 10) without changing any other parameters. As expected, the neuropathic model exhibited bursts whose frequency and duration increased with depolarization (**Fig. 5A**). An enlarged view of the membrane potential (**Fig. 5A** **inset**) shows the growth of MPOs preceding burst initiation, which has been observed experimentally [4], [7], [12], [35] and is a characteristic feature of elliptic bursting caused by the trajectory spiraling away from the fixed point until it jumps to the stable limit cycle. Noise was included in these simulations and introduces randomness into the initiation and termination of each burst, but it is unnecessary for bursting. On the other hand, bursting is dependent on the subcritical Hopf bifurcation; therefore, adding adaptation to the normal model predictably failed to produce bursting (data not shown).

**Figure 5B** explains the role of hysteresis in burst generation. The inset shows a typical burst with each phase of the response colored differently. Note that the burst starts and stops at different values of *z*, which is the activation variable for *I*_AHP_. The same response was projected onto a bifurcation diagram created by treating *z* as the bifurcation parameter rather than letting it evolve freely as a variable. The boundaries of the bistable region correspond to values of *z* at which the burst starts and stops. Fast-slow analysis can accurately predict whether adaptation will, for a certain stimulus intensity, lead to bursting or to tonic spiking at a reduced rate (**Fig. S3**).

Importantly, we did not tune parameters each time we sought to reproduce a different property; instead, MPOs and bursting were accurately reproduced in our model using the set of parameters chosen on the basis of spiking pattern in **Fig. 2**. Our results argue, therefore, (1) that spiking pattern, MPOs and bursting are mechanistically linked through their mutual dependence on the spike initiation mechanism, and (2) that our model captured the crucial nonlinearity (*i.e.* spike initiation) including how it is altered under neuropathic conditions.

### Continuum of injury-induced changes

**Figure 6:**
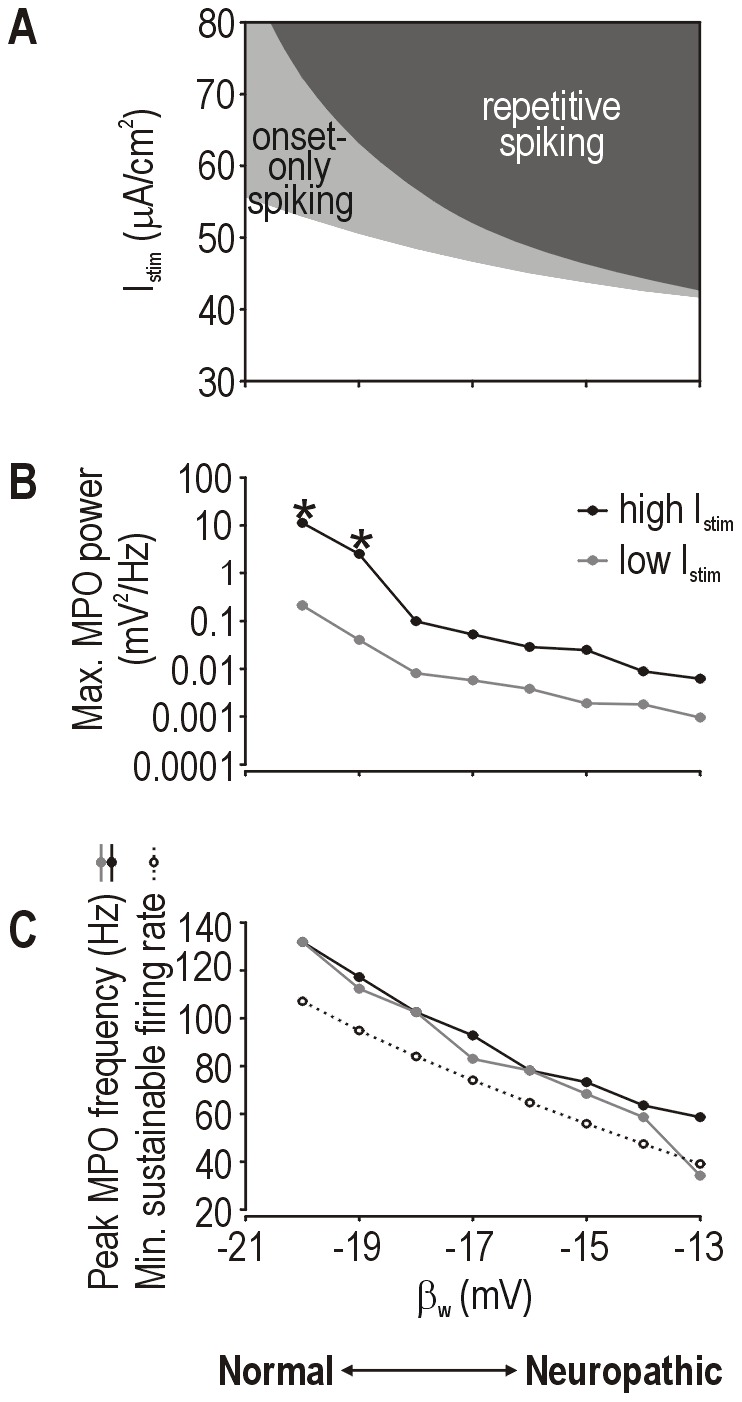
Simulating the continuum of pathological change. (**A**) Summary of *I*_stim_ thresholds to elicit onset-only or repetitive spiking for different values of β_w_ in our standard 2-D model. Reduction in threshold equates with an increase in excitability. Summary of peak MPO power (**B**) and peak frequency (**C**) across a range of β_w_ values. All simulations included noise. For each β_w_ value, *I*_stim_ was chosen relative to the threshold for repetitive spiking: *high* and *low I*_stim_ were 1.3 and 4 µA/cm^2^ below threshold, respectively. Those values were chosen in order to include or exclude, respectively, noise-independent MPOs when a supercritical Hopf bifurcation occurs. Peak MPO amplitude and frequency decreased as β_w_ was increased. That trend is not attributable to noise-independent MPOs occurring at certain β_w_ values since noise-independent MPOs were excluded when testing with *low I*_stim_ (see above). Moreover, re-setting γ_m_ from 18 mV to 15 mV prevented the supercritical Hopf bifurcation from occurring at any β_w_, but the same trend in MPO power and frequency was observed (data not shown). Dotted curve in C shows minimum sustainable firing rate. * indicates data points that include a noise-independent MPO component.

Beyond reproducing normal and neuropathic states, a good model should account for the transition between states by reproducing the continuum of pathological change. We are unaware of experimental data that quantitatively describe the progress of such changes, but one might reasonably assume that affected cells become *more* hyperexcitable as pathology worsens. Therefore, we tested values of β_w_ between the nominally “normal” value of −21 mV and the “neuropathic” value of −13 mV using our standard 2-D model. **Figure 6A** shows the progressive increase in excitability and the change in spiking pattern as β_w_ was varied. Specifically, the minimum *I*_stim_ required to elicit spiking decreased as β_w_ was increased, and the *I*_stim_ range associated with onset-only spiking became progressively narrower as repetitive spiking became predominant. Both trends are consistent with qualitative experimental data [4].

Next, we considered how MPOs changed within this same range of β_w_. All simulations included noise and *I*_stim_ was chosen to be just below the threshold for repetitive spiking. We found that MPO amplitude decreased as β_w_ was increased (**Fig. 6B**, black curve). Because intermediate values of β_w_ are associated with a supercritical Hopf bifurcation (see **Fig. 3B**) that gives rise to large, noise-independent MPOs (indicated by * on **Fig. 6B**) over a narrow range of *I*_stim_, we re-tested with *I*_stim_ further from threshold in order to isolate noise-dependent MPOs, but the same trend was observed (**Fig. 6B**, gray curve). Adjusting as few as one other parameter could prevent the supercritical Hopf bifurcation from occurring at any β_w_ value, but the same trend of decreasing MPO amplitude was still observed (data not shown). Peak MPO frequency also decreased over the same range of β_w_ (**Fig. 6C**, solid lines), which parallels the trend in minimal sustainable firing rate (dotted line) as expected from the type 2 excitability associated with the Hopf bifurcation [27], [36]. One might reasonably have expected MPOs to become larger and faster as pathology worsens, but the model clearly predicted the opposite. This unintuitive trend is explained by the neuron becoming more prone to repetitive spiking (see **Fig. 6A**) such that the large and fast MPOs observed after mild pathological change (*i.e.* small changes in β_w_) are replaced by repetitive spiking as pathology worsens (*i.e.* larger changes in β_w_); in other words, the upper limits of MPO amplitude and frequency are reduced as the lower limit (*i.e.* stimulus threshold) for repetitive spiking decreases.

Results presented thus far demonstrate that a switch in spike initiation mechanism (from QS-crossing to subcritical Hopf bifurcation) is sufficient to explain the complete triad of excitability changes outlined in **Fig. 1A–C**. Despite extensive exploration of the parameter space of two separate 2-D models (see also **Fig. S2**), we could not find another dynamical explanation for the combination of changes, consistent with the limited number of bifurcation mechanisms that are possible in a 2-D model [37], which argues that the identified switch is also necessary for the excitability changes. In a higher dimensional system, the same switch in spiking mechanism is sufficient to explain excitability changes but we cannot prove that it is necessary, although parsimony argues in favor of our explanation. We now shift our focus from what a switch in spike initiation can explain, to what explains the switch in spike initiation. Specifically, we will now consider the biophysical meaning of a neuropathic change in parameter β_w_ and whether such a change is biologically plausible. Later, we will consider the effects of changing other parameters.

### Biophysical basis for a neuropathic change in spike initiation

**Figure 7:**
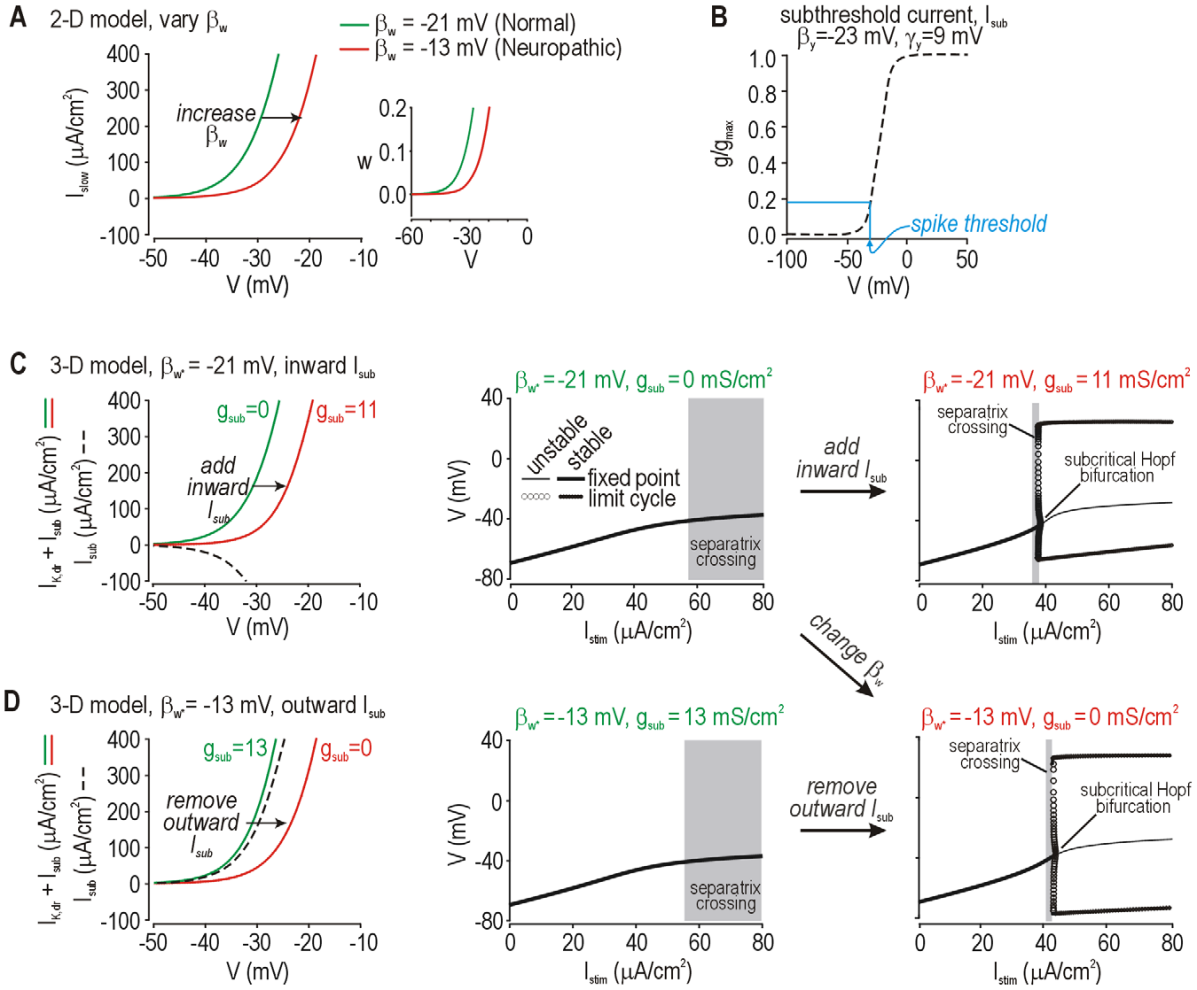
Relating parameter changes in the 2-D model with more biologically meaningful changes in a 3-D model. (**A**) Changing β_w_ from −21 mv to −13 mV shifts the *I*_slow_-*V* curve to the right, which corresponds to a rightward shift in the *w*-nullcline on the *V-w* phase plane (inset) and switches the spike initiation mechanism (see **Fig. 2**). In the 2-D model, β_w_ represents the voltage-dependency of *I*_slow_ which, in reality, comprises multiple currents with slow kinetics. The biological realism of the model can be increased by “ungrouping” *I*_slow_ into two (or more) components, one representing the delayed-rectifier potassium current *I*_K,dr_ and one representing a subthreshold current *I*_sub_ that can be inward or outward depending on the reversal potential. (**B**) Voltage-dependent activation curve for *I*_sub_. Parameter values (indicated on the figure) were determined as explained below. In the 3-D model, the (*I*_K,dr_+*I*_sub_)−*V* curve was shifted the same as the *I*_sub_-*V* curve in A by increasing inward *I*_sub_ (**C**) or by decreasing outward *I*_sub_ (**D**) on the basis of varying *g*_sub_. Bifurcation diagrams demonstrate the change in spike initiation mechanism. With *I*_K,dr_ properties fixed, maximal conductance and voltage-sensitivity of *I*_sub_ were adjusted to recreate the shift shown in A; derived parameters illustrate the importance of the modulated conductance activating at subthreshold potentials. Adding or removing the same subthreshold currents to a Hodgkin-Huxley model (rather than to our starting 2-D Morris-Lecar model) produces equivalent changes in excitability (data not shown). By comparison, modulating current that activates only at suprathreshold potentials (β_y_ = 0 mV) had no effect on the (*I*_K,dr_+*I*_supra_)−*V* curve in the perithreshold voltage range, and thus the spike initiation dynamics were unchanged (see **Fig. S4**).

Strictly speaking, β_w_ represents the voltage at half-maximal activation of the slow current, *I*_slow_ (see Eqn. 4). Increasing β_w_ from −21 mV to −13 mV caused a rightward shift in *I*_slow_-*V* curve of the model neuron (**Fig. 7A**), which corresponds to a shift in the *w*-nullcline (**Fig. 7A** **inset**); shifting the *w*-nullcline changes how the *V*- and *w*-nullclines intersect, which has direct implications for spike initiation dynamics (see **Fig. 2**). These results therefore argue that an injury-induced change in the voltage-dependency of *I*_slow_ could account for repetitive spiking; indeed, variations in the voltage-sensitivity of ion channel gating occur and are associated with hyperexcitability [e.g. 38], [39], [40]. However, most previous research has focused on whether expression of certain ion channels is up- or down-regulated after nerve injury [41]. Varying β_w_ can also account for changes in ion channel density if one understands how our “minimal” computer model was constructed; specifically, *I*_slow_ represents the sum of all ion currents with slow gating kinetics [42], meaning *I*_slow_ comprises multiple currents that could be modeled individually. To explain expression changes, we can ungroup *I*_slow_ into parts that have more specific molecular correlates. To this end, we replaced *I*_slow_ with a delayed rectifier K^+^ current *I*_K,dr_ and a subthreshold inward or outward current *I*_sub_ whose activation properties are shown in **Fig. 7B**. This converts the model from 2-D to 3-D, *i.e.* with three activation variables instead of just two (compare Eqns. 1 and 11). Increasing inward *I*_sub_ (akin to upregulating Na_v_1.3 expression [43]) or decreasing outward *I*_sub_ (akin to downregulating K_v_1 expression [44]) shifted the (*I*_K,dr_+*I*_sub_)−*V* curve in the 3-D model (**Fig. 7C and D**, respectively) the same way as making β_w_ less negative shifted the *I*_slow_-*V* curve in the 2-D model (cf. **Fig. 7A**); in other words, distinct molecular changes are equally capable of causing repetitive spiking, consistent with the equivalent effects of such changes on spike initiation (see bifurcation diagrams in **Fig. 7C and D**).

**Figure 8:**
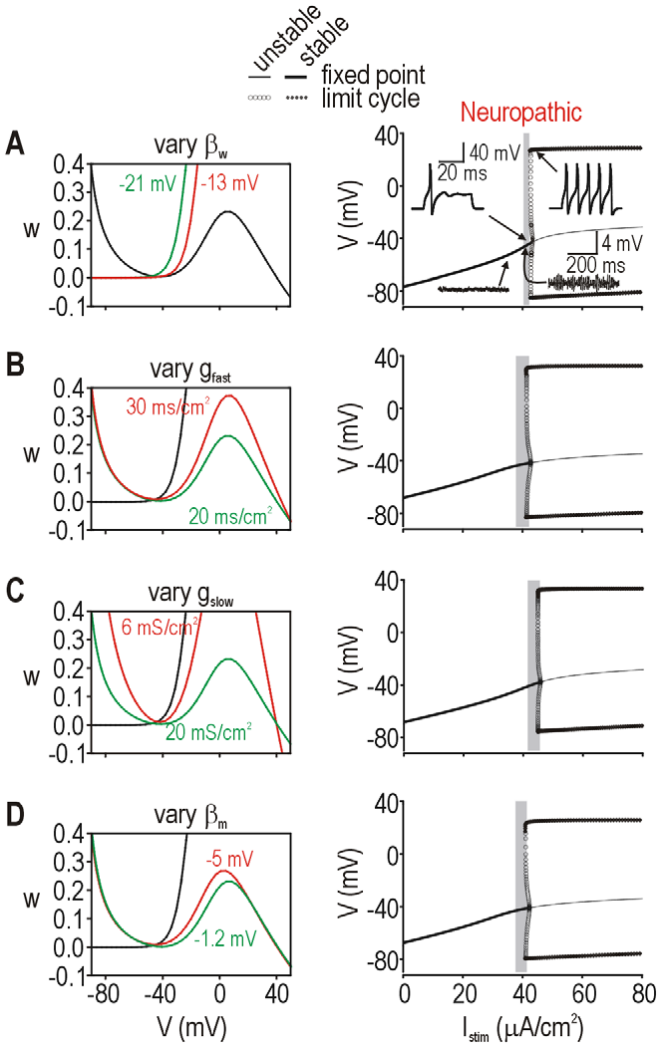
Dynamically equivalent effects of varying other model parameters in the 2-D model. Effects of changing β_w_ (**A**), *g*_fast_ (**B**), *g*_slow_ (**C**), and β_m_ (**D**) in the original 2-D model. All parameters were at their default values (see Methods) except for the parameter of interest, which was varied from its default value (green) to a value causing neuropathic excitability (red) as indicated on each panel. In each case, the shape and/or positioning of the *V*- or *w*-nullcline (shown on phase planes; left panels) was affected in a distinct way, but the geometry of the nullcline intersection showed the equivalent “neuropathic” change, as evidenced by the bifurcations diagrams (right panels); specifically, all “neuropathic” bifurcation diagrams exhibit a Hopf bifurcation. For A–D, the “normal” bifurcation diagrams are equivalent and correspond to that shown in **Fig. 2B** **top**.

These results demonstrate that a single parameter change in our minimal 2-D model can represent more than one biological change. Furthermore, other parameters in the 2-D model affect spike initiation. To investigate this, we varied other parameters one at a time and measured excitability. Starting from the same “normal” state (see **Fig. 2A** **top**), each parameter change was able to produce comparable “neuropathic” states as evidenced by a Hopf bifurcation (**Fig. 8A–D**, right panels). Phase planes (left panels) illustrate how different parameter changes morph either the *V*- or *w*-nullclines – the important observation is that each change produced the same geometrical alteration in how the two nullclines intersect, which is a direct reflection of how fast and slow currents compete during spike initiation (see **Fig. S1**). Like for the biological meaning of a change in β_w_ (see **Fig. 7**), we can reasonably posit that other parameter changes in the 2-D model can each represent more than one biological change.

**Figure 9:**
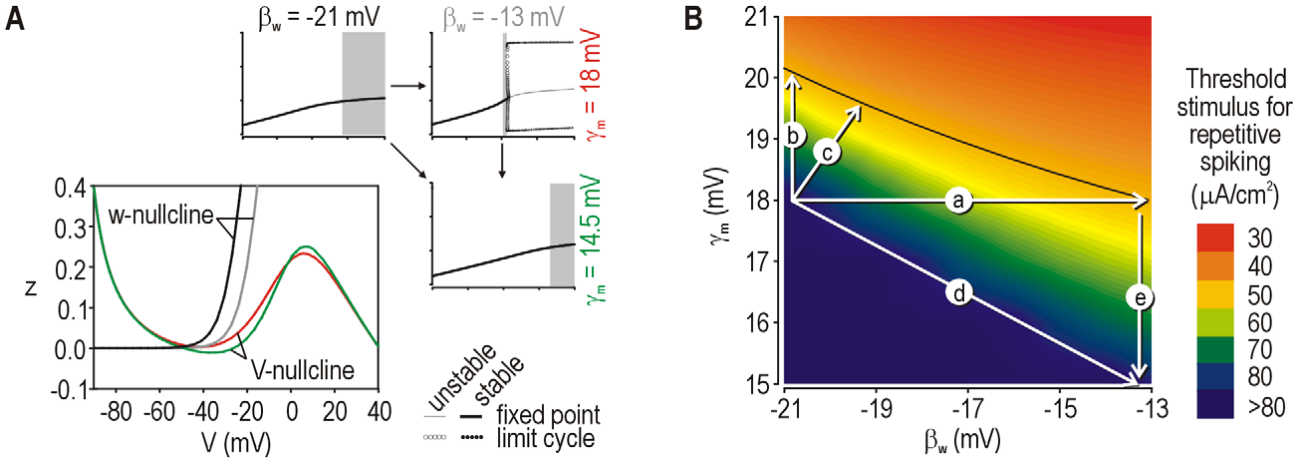
Effects of multiple co-occurring molecular changes. (**A**) A parameter change affecting a subthreshold current (in this case in the original 2-D model) may fail to cause hyperexcitability if that change is offset by a change in a second parameter. In this example, varying β_w_ shifts the *w*-nullcline whereas varying γ_m_ re-shapes the *V*-nullcline, but the combination of changes results in no change in the geometry of the nullcline intersection. Parameter values are indicated on the bifurcation diagrams; the color of each label corresponds to the color of nullclines shown on the *V-w* phase plane. (**B**) Changes in excitability (quantified as the *I*_stim_ threshold for repetitive spiking) caused by co-varying β_w_ and γ_m_. The increase in excitability caused by varying only β_w_ (arrow *a*) could be produced by a much smaller change in γ_m_ (arrow *b*) or by small combined changes in β_w_ and γ_m_ (arrow *c*). Arrow *d* shows conditions in A. Note that the reduction in γ_m_ required to offset a “neuropathic” change in β_w_ (arrow *e*) is larger than the increase in γ_m_ required to produce neuropathic excitability (arrow *b*). Systematic testing of all parameter combinations is beyond the scope of the current study, but this example highlights the importance of parameter co-variation.

These data emphasize the degeneracy of the molecular substrate for cellular excitability, *i.e.* that distinct molecular pathologies can yield the same pattern of cellular hyperexcitability (see Introduction). In fact, changing any parameter in the 2-D model can potentially produce the neuropathic change in spike initiation because our *minimal* model contains only the necessary and sufficient variables required to explain spike generation. If a conductance that does not influence spike initiation (e.g. one that activates only at suprathreshold potentials) is added to the 2-D model (thus producing a 3-D model comparable to that used in **Fig. 7**), altering that conductance will not affect excitability (**Fig. S4**). Notably, injury-induced changes in such currents could occur and would be correlated with cellular hyperexcitability (and neuropathic pain) without the molecular and cellular changes being causally related (see **Fig. 1D**). This illustrates the *insensitivity* of the pathological process to certain parameters [45]. On the other hand, a change in a functionally important parameter might also fail to produce cellular hyperexcitability if that change is offset by a simultaneous change in a second parameter (**Fig. 9A**). We illustrate this in **Fig. 9** using our 2-D model, but the importance of this observation is even greater for higher-dimensional models. Consider that biological changes accounted for by the same parameter might offset one another so that the parameter itself (in the 2-D model) does not change; for example, a large increase in inward *I*_sub_ could be offset by a large increase in outward *I*_sub_ (in the 3-D model) such that β_w_ remains unchanged (in the 2-D model). The last two examples highlight the importance of *co-variability* across parameters [45]. Notably, small co-variations may also combine to cause hyperexcitability (**Fig. 9B**). *Insensitivity* and *co-variability* are both routinely overlooked in studies reporting injury-induced changes in ion channel expression, thus compromising the definitive interpretation of those studies.

## Discussion

**Figure 10:**
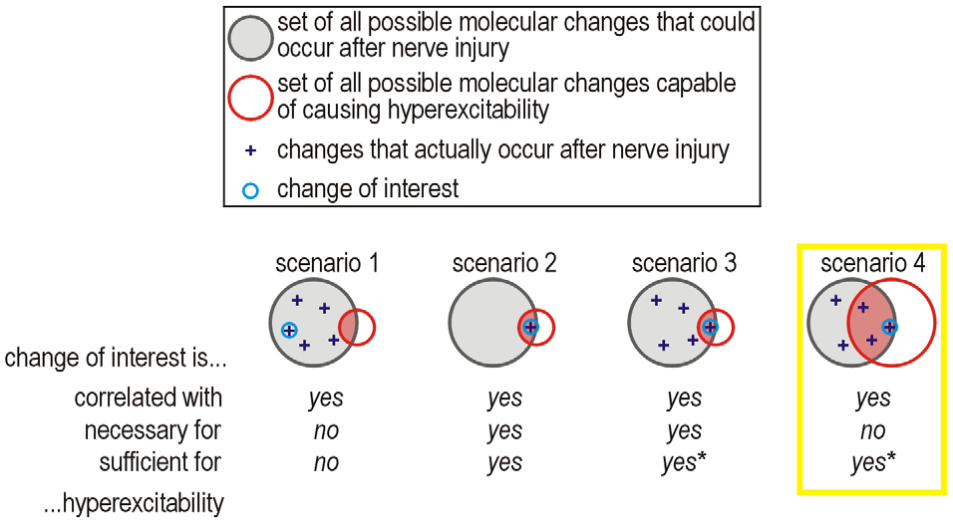
Summary of relationships between molecular and cellular changes. (**Scenario 1**) A molecular change of interest outside the red-shaded region is neither necessary nor sufficient to cause hyperexcitability, but may nevertheless be correlated with it. (**Scenario 2**) In the absence of other changes, a molecular change of interest inside the red-shaded region is both necessary and sufficient to cause hyperexcitability. (**Scenario 3**) If only one molecular change occurs inside the red-shaded region, that change will be necessary for hyperexcitability but may or may not be sufficient depending on how the change interacts with other changes outside the red-shaded region. (**Scenario 4**) If multiple molecular changes occur inside the red-shaded region, then the change of interest will not be necessary for hyperexcitability and may or may not be sufficient depending on how that change interacts with other changes. This last scenario (hightlighted in yellow) is the most likely given that nerve injury triggers multiple molecular changes and given the degenerate manner by which spike initiation can be altered, as shown in this study. Degeneracy implies that the red circle is large and thus likely to significantly overlap the gray circle.

In this study, we combined computer modeling and analysis based on dynamical systems theory to rigorously explain neuropathic changes in the excitability of primary somatosensory afferents. Beyond reproducing the quantitative increase in excitability, our results demonstrate that three qualitative changes in excitability (*i.e.* repetitive spiking, MPOs and bursting) all arise from a switch in the nonlinear mechanism responsible for spike initiation. In dynamical terms, spike initiation switches from a quasi-separatrix-crossing under normal conditions to a Hopf bifurcation under neuropathic conditions. These dynamical mechanisms represent different outcomes in the competition between positive and negative feedback, and generalize to different feedback mechanisms (*e.g.* Na^+^ channel inactivation vs. K^+^ channel activation). In biophysical terms, this switch occurs when competition between currents contributing to spike initiation becomes biased. A multitude of different changes in different channels are sufficient to produce the equivalent switch in spike initiation dynamics by biasing the competition in favor of inward current. A switch in spike initiation dynamics is thus sufficient (and very likely necessary) to explain neuropathic changes in excitability; by comparison, many different molecular changes may be sufficient to switch the spike initiation mechanism, but no single molecular change is strictly necessary if more than one sufficient change is triggered by nerve injury (see Introduction and **Fig. 10**).

Following a “top-down” modeling approach, we focused first on finding generic solutions to explain excitability changes, and only thereafter did we consider how such solutions might be biologically implemented. This contrasts a “bottom-up” approach of incorporating previously described injury-induced molecular changes into a computer model to investigate whether those molecular changes can account for changes in excitability. The two approaches are complementary but, prior to this study, the former had not been used to investigate primary afferent hyperexcitability, the implication being that no one has previously sought to specifically identify which molecular changes are sufficient to explain documented changes in excitability. Animal models of neuropathic pain can arguably recreate clinically relevant human conditions quite accurately, but such models exhibit a vast array of changes at multiple levels, which can make interpreting those changes prohibitively difficult, especially with respect to causality; irrelevant correlations are probably quite common (**Fig. 10** **scenario 1**). Our approach depends upon the empirical data collected in animal models of neuropathic pain (1) to identify cellular changes requiring explanation, and (2) to identify candidate molecular pathologies with which to explain those cellular changes. But with overwhelming amounts of such data already collected, an alternative approach such as ours is needed to help interpret those data.

Invariably, the question seems to arise: pathological alteration of which ion current is responsible (*i.e.* necessary) for hyperexcitability? Our results demonstrate that the answer is not straightforward (see **Fig. 10**). Any change in any current has context-dependent effects insofar as that altered current interacts with other currents. Indeed, a single mutation in Na_v_1.7 channels can increase or decrease excitability depending on the other channels present in the neuron [46]. Therefore, the more important issue is if and how interactions between currents are altered, and more specifically, whether that alteration manifests a switch in spike initiation dynamics. We would argue that one should ask: of all the changes caused by nerve injury, which are *sufficient* (alone or in combination) to produce cellular hyperexcitability? Experimentally testing the sufficiency of each change and combinations thereof for producing each aspect of excitability is impractical. Empirical testing in computer models is more feasible, but ideally a more strategic, theory-based approach could be employed. Our identification of spike initiation as the crucial nonlinearity linking simple molecular changes with complex cellular changes constrains the search to those currents affecting spike initiation. Only currents active at subthreshold potentials can contribute (directly) to the spike initiation process. *Supra*threshold currents like high-voltage-activated Ca_v_1 and 2 channels are present in somatosensory afferents and contribute to processes like synaptic transmission. Those currents can be altered by nerve injury [47] and such changes could contribute to neuropathic pain, but any association with primary afferent hyperexcitability is likely to be purely correlative and therapies targeting those currents would be ineffective at reversing hyperexcitability; however, the analgesic efficacy of gabapentenoid drugs acting on the α_2_-δ subunit of Ca_v_1 and 2 channels [48], [49] clearly implicates other mechanisms as critical factors for pain processing. But even if primary afferent hyperexcitability is neither necessary nor sufficient to produce neuropathic pain (see Introduction), primary afferent hyperexcitability is an important contributing factor [3]. Our data highlight the nuances and complicated nature of the molecular basis for just this one contributing factor.

Although we have ruled in subthreshold currents as playing a critical role in spike initiation and excitability, it cannot be assumed that even large changes in a *sub*threshold current will necessarily lead to cellular hyperexcitability, let alone neuropathic pain. Cellular excitability may or may not be affected depending on concurrent changes in other currents that could offset the first change (**Fig. 10** **, scenarios 3 and 4**). This raises an important point: most studies have focused on characterizing injury-induced changes in one channel, or perhaps a few channels, but a definitive explanation of whether such changes are sufficient to cause cellular hyperexcitability requires one to account for co-variations by measuring expression levels of all channels involved in spike initiation. Worse yet, this complete analysis should be done on a cell-by-cell basis since different neurons may achieve equivalent excitability based on different combinations of channel densities [50], [51], [52]. Beyond expression levels, we must also consider that channel function can be modulated [53].

In terms of developing new analgesics that are more effective against neuropathic pain, it is arguably less important to understand precisely which molecular changes combine to cause hyperexcitability than it is to understand which molecular processes should be targeted when trying to therapeutically reverse hyperexcitability. In other words, one need not reverse underlying molecular changes if cellular excitability can be normalized via other (potentially more druggable) targets. By this logic, one should focus on the sufficiency (and likely necessity) of a dynamical change in spike initiation for explaining hyperexcitability rather than being preoccupied with the degeneracy of the molecular basis for that switch. Indeed, whereas the diversity of molecular changes contributing to hyperexcitability makes it difficult to understand the exact pathogenic process (see above), that same diversity broadens the range of drug targets whereby hyperexcitability might be corrected. However, degeneracy of the molecular substrate for cellular excitability has another important implication: if neuropathic pain results from maladaptive plasticity [20], [54], then the therapeutic efficacy achieved through effects on one ion channel could be offset (*i.e.* nullified) by misguided homeostatic plasticity in any one of several other ion channels. This might help explain the relative intractability of neuropathic pain, and suggests that we must be prepared to block or reverse changes in each and every ion channel affected by maladaptive homeostatic plasticity or, better yet, that we address the homeostatic plasticity rules themselves rather than the diverse agents upon which those rules act. The diversity of molecular pathologies contributing to neuropathic pain is arguably well recognized, but the implications described above are not. Our results caution against that oversight.

Our results also demonstrate how simple molecular changes can manifest complex changes in cellular excitability because of how molecular processes interact. The nonlinear nature of those interactions is key for enabling small quantitative changes in the interacting processes to cause large qualitative changes in the outcome. Indeed, this is the case for spike initiation insofar as there is a critical point (defined by a balance of time- and voltage-dependent feedback mechanisms) that determines whether an all-or-none spike will be produced. Notably, if a simple conductance-based model can accurately reproduce phenomena of interest, then it is necessarily true that a more complicated, biologically realistic model (*i.e.* with more variables and parameters) will be able to do the same. That said, finding the required set of parameter values within the larger parameter space of the more complicated model may prove prohibitively difficult. Nonetheless, either type of model could be used to study the phenomena – the choice of model depends on the specific questions and how one intends to answer them. In our case, we started with a simple model because we sought to rigorously characterize (pathological disruption of) fundamental mechanisms of excitability using tools like phase-plane analysis. Once that characterization was complete, we increased the biological realism of our model (see **Fig. 7**) to facilitate its biological interpretation. Our results demonstrate the utility of such an approach.

To summarize, our results demonstrate that a switch in spike initiation mechanism is sufficient and most likely necessary to explain a constellation of neuropathic changes in primary afferent excitability. Pathological alteration of the dynamical mechanism responsible for spike initiation, rather than pathological alteration of any one ion channel, is key for explaining cellular hyperexcitability. Indeed, the molecular basis for a change in spike initiation dynamics is highly degenerate insofar as a multitude of different molecular changes, either alone or in combination, can manifest the same change in cellular excitability. Ultimately, strategically intervening to reduce neuropathic pain requires that we thoroughly understand and capitalize on rather than be thwarted by the complexity and degeneracy of the underlying mechanisms.

## Methods

### Two-dimensional model

Simulations were conducted in modified versions of the Morris-Lecar model. All models are single compartment, which is important for enabling our analysis but is also an accurate representation of acutely isolated somata used in most electrophysiological experiments on dorsal root ganglion neurons, including those reproduced in **Fig. 1**. The simplest, 2-D version of our model consists of a fast activation variable *V* and a slower recovery variable *w* [27], [33], [55]. *V* denotes membrane potential and *m* and *w* are gating variables.
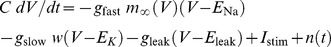
(1)

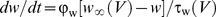
(2)

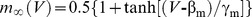
(3)

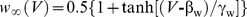
(4)

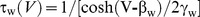
(5)such that *m* changes instantaneously with *V* whereas *w* changes with a time constant τ_w_. Unless otherwise indicated, parameters were *C* = 2 µF/cm^2^, *E*_Na_ = 50 mV, *E*_K_ = −100 mV, *E*_leak_ = −70 mV, ϕ_w_ = 0.15, *g*_fast_ = 20 mS/cm^2^, *g*_slow_ = 20 mS/cm^2^, *g*_leak_ = 2 mS/cm^2^, β_m_ = −1.2 mV, γ_m_ = 18 mV, γ_w_ = 10 mV, and β_w_ was varied. These values were taken directly from [27] except that β_w_ was freely adjusted in **Fig. 2** in order to reproduce different spiking patterns; thereafter, parameter values were *not* adjusted to reproduce other response properties. *I*_stim_ represents injected current. Where indicated, Gaussian white noise *n*(*t*) with 0 mean and 20 mV^2^ variance was added. This weak noise approximates effects of stochastic channel opening. DRG somata do not experience synaptic noise because they do not bear any synapses.

### Three-dimensional model with slow sodium channel inactivation

Slow Na^+^ channel inactivation was incorporated into the 2-D model by inserting an inactivation variable *h* according to
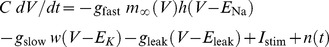
(6)

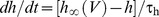
(7)

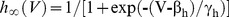
(8)where β_h_ = 30 mV, γ_h_ = −5 mV, τ_h_ = 2000 ms, γ_m_ = 15 mV, and all other parameters were unchanged from our standard 2-D models.

### Three-dimensional model with adaptation

The model with adaptation mediated by an AHP current *I*_AHP_ [56] was modeled according to
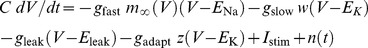
(9)

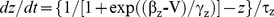
(10)where *z* controls activation of *I*_AHP_ current with parameters g_adapt_ = 0.5 mS/cm^2^, β_z_ = 0 mV, γ_z_ = 4 mV, τ_z_ = 300 ms. All other parameters were unchanged from our standard 2-D models.

### Three-dimensional “ungrouped” model

In the standard 2-D model, *g*_fast_ and *g*_slow_ represent the sum of all conductances with fast or slow activation kinetics, respectively. To help interpret the biophysical meaning of changing β_w_ in the 2-D model (see Results), we ungrouped *I*_slow_ into a delayed rectifier K^+^ current *I*_K,dr_ and a subthreshold current *I*_sub_ that is either inward or outward depending on *E*_sub_. Activation of *I*_K,dr_ and *I*_sub_ are controlled by w* and *y*, respectively, as described by
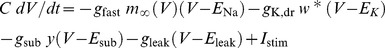
(11)where differential equations for w* and *y* are the same form as Eqn. 2, and steady state values and time constants are calculated as per Eqns. 4 and 5 with parameters ϕ_w*_ = 0.15, β_w*_ = −21 mV or −13 mV, γ_w*_ = 10 mV, ϕ_y_ = 0.3, E_sub_ = E_Na_ = 50 mV for inward current and ϕ_y_ = 0.15, E_sub_ = E_K_ = −100 mV for outward current. Values of *g*_sub_, β_y_ and γ_y_ were adjusted to reproduce effects of varying β_w_ in the 2-D model and are indicated in the legend of **Fig. 7**. All other parameters were as in the 2-D model.

### Simulation methods and dynamical analysis

Simulations were run in XPP [57] using the Euler method with 0.01–0.05 ms time step. Phase plane analysis was conducted in XPP and bifurcation analysis was conducted with AUTO using the XPP interface. Excellent introductions to these forms of nonlinear dynamical analysis are available in [26], [33], [34], [37]. In **Fig. 2C**, the quasi-separatrix (QS) was plotted on the *V-w* phase plane by integrating backward in time starting from the point indicated by *. That point was chosen based on where forward trajectories (with slightly different starting values) eventually diverge after clinging to the QS. The region of divergence marks the end of the QS and is therefore a good location from which to begin plotting the reverse trajectory representing the QS.

## Supporting Information

Figure S1 **Spike initiation dynamics represent competition between feedback mechanisms.** Spike initiation depends on competition between fast-activating, net inward current (a positive feedback process) and slower-activating, net outward current (a negative feedback process). A spike is initiated when positive feedback proceeds unchecked by negative feedback. Fast current comprises *I*_fast_, *I*_leak_ and *I*_stim_. Slow current comprises only *I*_slow_. (**A**) Sample responses from the normal and neuropathic models to just-suprathreshold stimulation (starting at *t* = 0 ms with *I*_stim_ = 60 and 45 µA/cm^2^, respectively). Insets labeled *a*–*d* show enlarged views during successful or unsuccessful spike initiation. Arrows mark where *dV*/*dt* starts to increase because of runaway positive feedback. In the normal model (top), inward and outward current are both strongly activated and the former just wins (*a*); after one spike, outward current settles at a new steady-state that is sufficiently strong to prevent further spiking (*b*). In the neuropathic model, inward current starts activating with relatively little counterbalancing response from outward current during the first and later spikes (c, *d*). The competition can also be visualized by plotting currents against each other (**B**) rather than against time, as in A. Right graph shows enlarged view of yellow region on left graph and highlights the spike initiating phase. Labels *a–d* correspond to those in A. In normal conditions (green), the steep trajectory labeled *a* indicates that outward current, despite its slower kinetics, almost manages to counterbalance fast-activating inward current, and in fact this does occur in the failed spike labeled *b*. In neuropathic conditions (red), the shallower trajectories labeled *c* and *d* show that competition has become biased in favor of inward current. The difference between trajectories suggests that slow-activating outward current is relatively weaker or that fast-activating inward current is relatively stronger under neuropathic conditions. To summarize, in the normal model, fast-activating inward current only ever “wins” at short latencies after stimulus onset, before slower-activating outward current reaches its new steady state; at steady state, outward current is sufficient to stabilize the system and thereby prevent further spiking. In contrast, in the neuropathic model, steady state outward current is insufficient to counterbalance fast-activating inward current when stimulation exceeds a critical intensity, and repetitive spiking ensues.
(TIF)

Figure S2
**Spike initiation in model with Na^+^ channel inactivation instead of K^+^ channel activation.** In our standard 2-D model (see Eqns. 1–5), spikes are generated on the basis of competition between fast-activating Na^+^ current and slower-activating K^+^ current. These two processes represent fast positive feedback and slow negative feedback, respectively. Slow negative feedback can also be mediated by sodium channel inactivation, according to
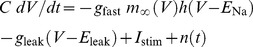
(S1)

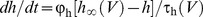
(S2)

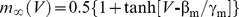
(S3)

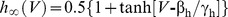
(S4)

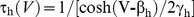
(S5)where *h* controls inactivation. Eqn. S1, S2, S3, S4, S5 are essentially equivalent to Eqn. 1–5. Parameters were the same as in our standard 2-D model except for the following: β_m_ = −5 mV, γ_m_ = 15 mV, β_h_ = −27 mV or −30 mV and γ_h_ = −8 mV. Notably, the Na^+^ channel inactivation modeled here is much faster than that modeled in Eqn. 7 and 8, but *h* nevertheless changes slowly relative to activation *m*. (**A**) The model with β_h_ = −30 mV exhibited onset-only spiking generated through a QS-crossing and negligible MPOs over a broad range of *I*_stim_. Compare with top row of **Figure 2**. Orientations of the *V*- and *h*-nullclines differ from those in **Figure 2**, but both nullclines are inverted such that their intersection with each other is unchanged. (**B**) The model with β_h_ = −27 mV exhibited repetitive spiking generated through a subcritical Hopf bifurcation and sizeable MPOs as the stable fixed point neared instability. Compare with bottom row of **Figure 2**. These data demonstrate that regardless of exactly how the model is constructed, spike initiation depends on competition between fast positive feedback and slower negative feedback. Neuropathic changes in excitability represent a qualitative change in the outcome of that competition.(TIF)

Figure S3 **Fast-slow analysis of adaptation.** (**A**) Sample responses to stimulation with *I*_stim_ = 43 µA/cm^2^ (left) and 46 µA/cm^2^ (right) in the 3-D neuropathic model (β_w_ = −13 mV) with adaptation. Bifurcation analysis of the fast subsystem was conducted by systematically varying *z*, like in Figure 5; those results are shown with black and gray curves in B and C. In **B**, we overlaid the *z*-nullcline (green) and *V*_equivalent_ (red), which corresponds to the voltage that would produce adaptation equivalent to the average adaptation within one inter-spike interval, where that interval is a function of *z* [see ref. S1 for details]. For both stimulus intensities, the *z*-nullcline does not intersect the stable-fixed-point-branch of the fast subsystem, which predicts that adaptation will not stabilize the cell in a quiescent state [ref. S2]. By comparison, for strong stimulation, the *z*-nullcline intersects the stable branch of *V*_equivalent_ (*), which predicts that adaptation will stabilize at that intersection point, resulting in tonic spiking at a fixed rate, whereas for weak stimulation, the *z*-nullcline intersects the unstable branch of *V*_equivalent_ (inset), which predicts that adaptation will not stabilize, thus resulting in bursting. In the latter case, *z* increases toward a value which, if it could be reached, would stabilize the neuron at a tonic firing rate, but spiking stops before that value is reached, at which point *z* falls until spiking resumes – repeated unsuccessful attempts to reach this unattainable value of *z* causes bursting. In **C**, responses from the 3-D model (same as in A) are projected onto the bifurcation diagrams, and confirm the predictions explained in B. S1. Golomb D, Yue C, Yaari Y (2006) Contribution of persistent <1?ri?>Na+ current and M-type K+ current to somatic bursting in CA1 pyramidal cells: combined experimental and modeling study. J Neurophysiol 96: 1912–1926. S2. Prescott SA, Sejnowski TJ (2008) Spike-rate coding and spike-time coding are affected oppositely by different adaptation mechanisms. J Neurosci 28: 13649–13661.
(TIF)

Figure S4 **Changes in suprathreshold currents fail to cause hyperexcitability.** By adding an additional current to our 2-D model, we produced a 3-D model comparable to that described in Figure 7. (**A**) Voltage-dependent activation curve for suprathreshold current *I*_supra_. Compare with activation curve for *I*_sub_ in Figure 7B. (**B**) Adding *I*_supra_ did not shift the (*I*_K,dr_+*I*_supra_)−*V* curve in the voltage range near spike threshold. Bifurcation analysis (right) confirmed that there was no change spike initiation mechanism and numerical simulations (not shown) confirmed that there was no change in spiking pattern, MPOs or bursting, although spike width was markedly increased. Predictably, there was also no change in the nullcline geometry (not shown).
(TIF)
