# Supplementary figures and images for: Identification of Molecular Pathologies Sufficient to Cause Neuropathic Excitability in Primary Somatosensory Afferents Using Dynamical Systems Theory
Source: PLoS Comput Biol. 2012 May 24;8(5):e1002524. doi: 10.1371/journal.pcbi.1002524 (PMC3359967; doi:10.1371/journal.pcbi.1002524)

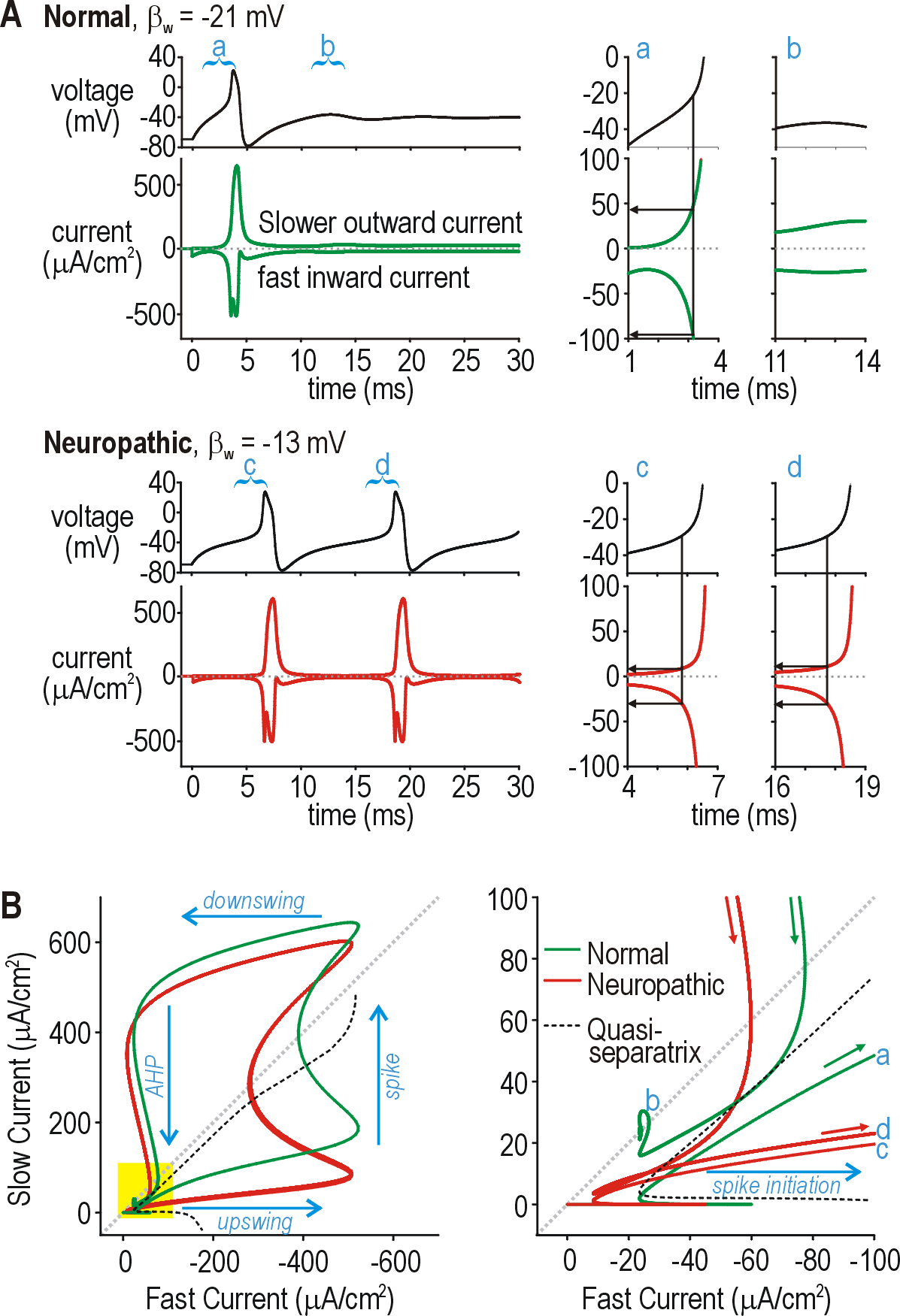

Supplement: Figure S1 — Spike initiation dynamics represent competition between feedback mechanisms. Spike initiation depends on competition between fast-activating, net inward current (a positive feedback process) and slower-activating, net outward current (a negative feedback process). A spike is initiated when positive feedback proceeds unchecked by negative feedback. Fast current comprises I fast, I leak and I stim. Slow current comprises only I slow. (A) Sample responses from the normal and neuropathic models to just-suprathreshold stimulation (starting at t = 0 ms with I stim = 60 and 45 µA/cm2, respectively). Insets labeled a–d show enlarged views during successful or unsuccessful spike initiation. Arrows mark where dV/dt starts to increase because of runaway positive feedback. In the normal model (top), inward and outward current are both strongly activated and the former just wins (a); after one spike, outward current settles at a new steady-state that is sufficiently strong to prevent further spiking (b). In the neuropathic model, inward current starts activating with relatively little counterbalancing response from outward current during the first and later spikes (c, d). The competition can also be visualized by plotting currents against each other (B) rather than against time, as in A. Right graph shows enlarged view of yellow region on left graph and highlights the spike initiating phase. Labels a–d correspond to those in A. In normal conditions (green), the steep trajectory labeled a indicates that outward current, despite its slower kinetics, almost manages to counterbalance fast-activating inward current, and in fact this does occur in the failed spike labeled b. In neuropathic conditions (red), the shallower trajectories labeled c and d show that competition has become biased in favor of inward current. The difference between trajectories suggests that slow-activating outward current is relatively weaker or that fast-activating inward current is relatively stronger under [file pcbi.1002524.s001.tif]

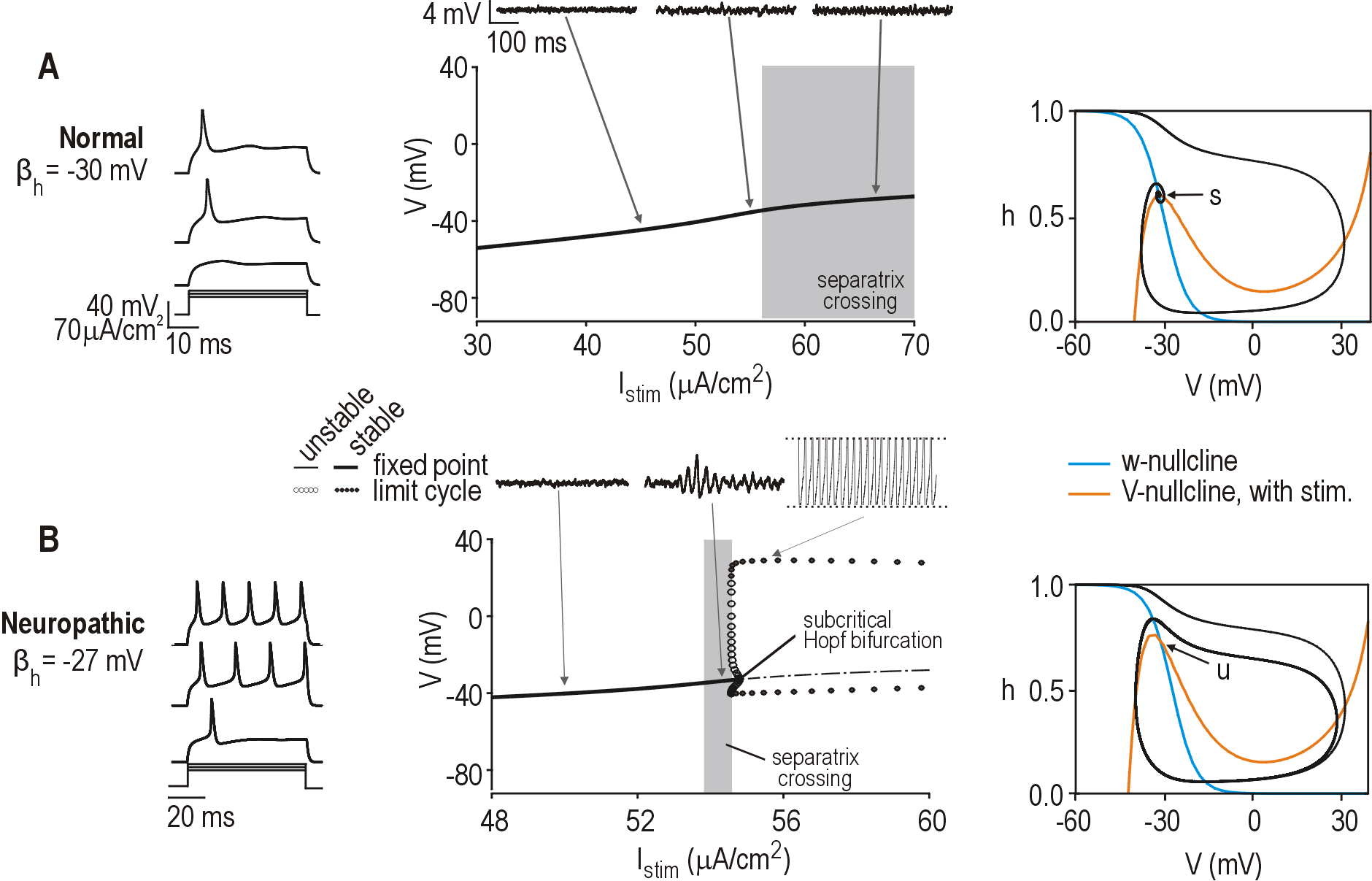

Supplement: Figure S2 — Spike initiation in model with Na+ channel inactivation instead of K+ channel activation. In our standard 2-D model (see Eqns. 1–5), spikes are generated on the basis of competition between fast-activating Na+ current and slower-activating K+ current. These two processes represent fast positive feedback and slow negative feedback, respectively. Slow negative feedback can also be mediated by sodium channel inactivation, according to(S1) (S2) (S3) (S4) (S5)where h controls inactivation. Eqn. S1, S2, S3, S4, S5 are essentially equivalent to Eqn. 1–5. Parameters were the same as in our standard 2-D model except for the following: βm = −5 mV, γm = 15 mV, βh = −27 mV or −30 mV and γh = −8 mV. Notably, the Na+ channel inactivation modeled here is much faster than that modeled in Eqn. 7 and 8, but h nevertheless changes slowly relative to activation m. (A) The model with βh = −30 mV exhibited onset-only spiking generated through a QS-crossing and negligible MPOs over a broad range of I stim. Compare with top row of Figure 2 . Orientations of the V- and h-nullclines differ from those in Figure 2 , but both nullclines are inverted such that their intersection with each other is unchanged. (B) The model with βh = −27 mV exhibited repetitive spiking generated through a subcritical Hopf bifurcation and sizeable MPOs as the stable fixed point neared instability. Compare with bottom row of Figure 2 . These data demonstrate that regardless of exactly how the model is constructed, spike initiation depends on competition between fast positive feedback and slower negative feedback. Neuropathic changes in excitability represent a qualitative change in the outcome of that competition. (TIF) [file pcbi.1002524.s002.tif]

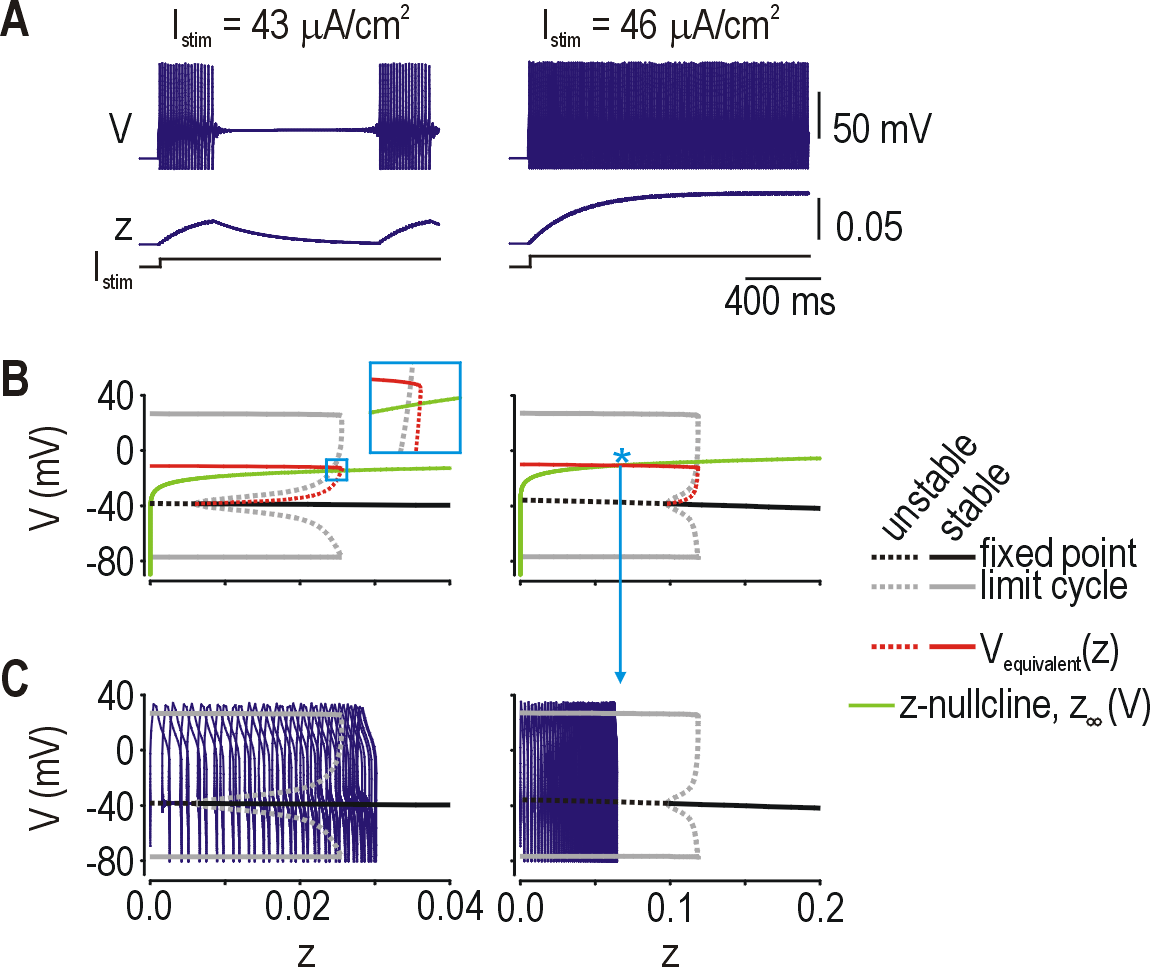

Supplement: Figure S3 — Fast-slow analysis of adaptation. (A) Sample responses to stimulation with I stim = 43 µA/cm2 (left) and 46 µA/cm2 (right) in the 3-D neuropathic model (βw = −13 mV) with adaptation. Bifurcation analysis of the fast subsystem was conducted by systematically varying z, like in Figure 5; those results are shown with black and gray curves in B and C. In B, we overlaid the z-nullcline (green) and V equivalent (red), which corresponds to the voltage that would produce adaptation equivalent to the average adaptation within one inter-spike interval, where that interval is a function of z [see ref. S1 for details]. For both stimulus intensities, the z-nullcline does not intersect the stable-fixed-point-branch of the fast subsystem, which predicts that adaptation will not stabilize the cell in a quiescent state [ref. S2]. By comparison, for strong stimulation, the z-nullcline intersects the stable branch of V equivalent (*), which predicts that adaptation will stabilize at that intersection point, resulting in tonic spiking at a fixed rate, whereas for weak stimulation, the z-nullcline intersects the unstable branch of V equivalent (inset), which predicts that adaptation will not stabilize, thus resulting in bursting. In the latter case, z increases toward a value which, if it could be reached, would stabilize the neuron at a tonic firing rate, but spiking stops before that value is reached, at which point z falls until spiking resumes – repeated unsuccessful attempts to reach this unattainable value of z causes bursting. In C, responses from the 3-D model (same as in A) are projected onto the bifurcation diagrams, and confirm the predictions explained in B. S1. Golomb D, Yue C, Yaari Y (2006) Contribution of persistent <1?ri?>Na+ current and M-type K+ current to somatic bursting in CA1 pyramidal cells: combined experimental and modeling study. J Neurophysiol 96: 1912–1926. S2. Prescott SA, Sejnowski TJ (2008) Spike-rate coding and spike-time coding are affected oppositely b [file pcbi.1002524.s003.tif]

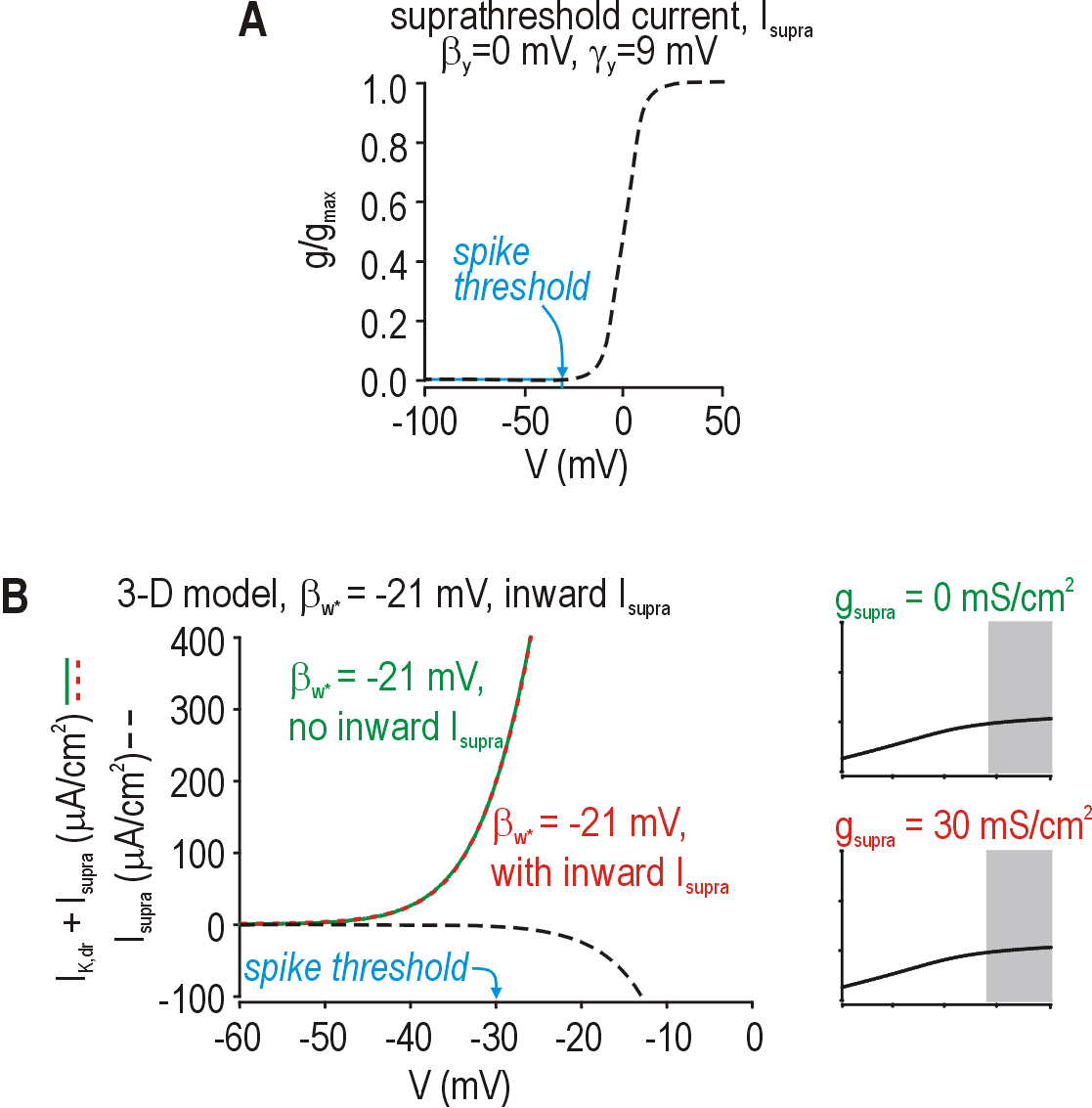

Supplement: Figure S4 — Changes in suprathreshold currents fail to cause hyperexcitability. By adding an additional current to our 2-D model, we produced a 3-D model comparable to that described in Figure 7. (A) Voltage-dependent activation curve for suprathreshold current I supra. Compare with activation curve for I sub in Figure 7B. (B) Adding I supra did not shift the (I K,dr+I supra)−V curve in the voltage range near spike threshold. Bifurcation analysis (right) confirmed that there was no change spike initiation mechanism and numerical simulations (not shown) confirmed that there was no change in spiking pattern, MPOs or bursting, although spike width was markedly increased. Predictably, there was also no change in the nullcline geometry (not shown). (TIF) [file pcbi.1002524.s004.tif]
